# Substantial Increases Occur in Serum Activins and Follistatin during Lung Transplantation

**DOI:** 10.1371/journal.pone.0140948

**Published:** 2016-01-28

**Authors:** David M. de Kretser, Jonathan G. Bensley, David J. Phillips, Bronwyn J. Levvey, Greg I. Snell, Enjarn Lin, Mark P. Hedger, Robyn E. O’Hehir

**Affiliations:** 1 Department of Anatomy and Developmental Biology, School of Biomedical Sciences, Monash University, Clayton, Victoria, Australia; 2 Hudson Institute of Medical Research, Clayton, Victoria, Australia; 3 Department of Allergy, Immunology and Respiratory Medicine, Alfred Hospital, Melbourne, Victoria, Australia; 4 Lung Transplant Service, Alfred Hospital, Melbourne, Victoria, Australia; 5 Central Clinical School, Monash University, Melbourne, Victoria, Australia; 6 Department of Anaesthesia and Perioperative Medicine, Alfred Hospital, Melbourne, Victoria, Australia; McGill University, CANADA

## Abstract

**Background:**

Lung transplantation exposes the donated lung to a period of anoxia. Re-establishing the circulation after ischemia stimulates inflammation causing organ damage. Since our published data established that activin A is a key pro-inflammatory cytokine, we assessed the roles of activin A and B, and their binding protein, follistatin, in patients undergoing lung transplantation.

**Methods:**

Sera from 46 patients participating in a published study of remote ischemia conditioning in lung transplantation were used. Serum activin A and B, follistatin and 11 other cytokines were measured in samples taken immediately after anaesthesia induction, after remote ischemia conditioning or sham treatment undertaken just prior to allograft reperfusion and during the subsequent 24 hours.

**Results:**

Substantial increases in serum activin A, B and follistatin occurred after the baseline sample, taken before anaesthesia induction and peaked immediately after the remote ischemia conditioning/sham treatment. The levels remained elevated 15 minutes after lung transplantation declining thereafter reaching baseline 2 hours post-transplant. Activin B and follistatin concentrations were lower in patients receiving remote ischemia conditioning compared to sham treated patients but the magnitude of the decrease did not correlate with early transplant outcomes.

**Conclusions:**

We propose that the increases in the serum activin A, B and follistatin result from a combination of factors; the acute phase response, the reperfusion response and the use of heparin-based anti-coagulants.

## Introduction

Lung transplantation enables patients to survive otherwise fatal pulmonary diseases [1, 2], but a shortage of donors causes long waiting lists. Hence, minimizing graft damage and optimizing transplantation outcomes are critical. Ischemia reperfusion injury (IRI), increased graft immunogenicity, delayed graft function, acute rejection, and the subsequent development of chronic allograft dysfunction contribute to graft loss [3–5]. Optimizing graft quality by improving donor conditioning, better preservation media, and prevention of transplant damage during surgery will enhance transplant outcomes [6]. Despite these approaches, IRI results in primary graft dysfunction, the leading cause of early morbidity and mortality after lung transplantation [7, 8]. Paradoxically, transplant reperfusion initiates events culminating in graft inflammation by up-regulating agents that promote neutrophil adhesion, mononuclear cell organ infiltration and secretion of T-cell associated cytokines, all contributing to IRI [3–5, 9, 10].

The role of the activins, members of the transforming growth factor-β(TGFβ) superfamily, in inflammation emerged from the rapid increase in serum activin A after a lipopolysaccharide(LPS) challenge in mice [11–13]. This activin A response is induced by signalling via Toll-Like Receptor 4(TLR4) [13] and stimulates secretion of follistatin (FS), a high affinity (K_d_ 50-900pM) activin binding protein. The resulting activin-FS complex is targeted to a lysosomal degradation pathway [14, 15]. Inflammation-induced Activin A also initiates fibrosis in hepatic and renal injury models [16, 17]. Furthermore, activin A also increases as part of the acute phase response to surgery [18].

Alternative gene splicing results in secretion of two forms of follistatin, FS315 (315 amino acids) and FS288 (288 amino acids), the latter binds to negatively charged cell surface heparan sulphate (HS) containing proteoglycans [15, 19]. Consequently, administration of negatively charged heparin releases both activin and FS from cell surfaces [20–22]. FS288 is the more potent isoform with a substantially longer biological action than FS315 [19]. Reperfusion after ischemic cardiac damage also causes local activin A and FS production which decreases cardiomyocyte mitochondrial membrane potential and associated damage that can be ameliorated by follistatin [23].

Since transplantation surgery uses heparin and IRI induces an inflammatory response, we determined the serum activin A, B and FS responses in serum samples from patients who participated in a previously published randomized study of remote ischemic conditioning (RIC) during lung transplantation [24]. While that study identified a trend toward a decrease in primary graft dysfunction in patients receiving RIC, significance was only achieved in patients with a diagnosis of restrictive lung disease. That study also defined a peak in the serum levels of some cytokines and the high molecular weight group box 1 protein within 2 hours after lung reperfusion.

## Ethics, Study Design, and Methods

### Ethics

This study was approved by the Alfred Hospital Human Research Ethics Committee (Approval number: 1/09/0257; Australian New Zealand Clinical Trials Registration Number: ACTRN12609000763246). Consent was provided in writing. None of the transplant donors were from a vulnerable population and all donors or next of kin provided informed consent that was freely given.

### Study Design

Serum samples from 46 of 60 patients participating in the prospective, parallel arm randomized controlled trial of RIC in patients, 18 years or older, undergoing bilateral sequential lung transplantation were used. They were stratified by diagnosis (restrictive disease, cystic fibrosis (CF) or non-CF) [24] The RIC involved 3 cycles of lower limb ischemic conditioning performed just prior to the reperfusion of the first lung transplant. Patients receiving extra-corporeal membranous oxygen and/or cardio-pulmonary bypass after surgery were excluded from the study, as there were too few to reliably estimate the effects of extra-corporeal membranous oxygen and/or cardio-pulmonary bypass on activin A, activin B, follistatin or the other cytokines we examined.

### Transplantation

Lungs retrieved from donations both after brain or cardiac death, were preserved by a single ante-grade flush using Perfadex (Vitrolife, Goteborg, Sweden). All patients received immunosuppression with tacrolimus and azathioprine pre-operatively and methyl prednisolone (1 gram) intra-operatively (500 mg at both anaesthetic induction, and first donor lung reperfusion). Patients also received 2500–5000 IU boluses of heparin to maintain activated clotting times at twice baseline with an average total dose 5000 IU of heparin per patient.

### Sample collection and immunoassays

Blood samples were obtained just after anaesthesia induction, after RIC or sham treatment, and 15 minutes, 2 hours, 8 hours and 24 hours after lung reperfusion. After clotting, serum was separated and stored frozen until used. Total serum activin A, B and FS were measured by ELISA and by specific radioimmunoassay, respectively, with normal ranges based on samples from 138 normal males and females [25, 26] providing a reference maximum (RM). The other cytokines were measured using the Human Th1/Th2 CBA Kit II and the Human Chemokine CBA Kit (BD Biosciences, Mountain View, California, USA), with data acquired on an LSRII cytometer and analysed using FCAP Array Software (BD Biosciences).

### Outcomes

Primary outcome measures were serum levels of activin A, activin B, FS, interleukin 2 (IL2), interleukin 4 (IL4), interleukin 6 (IL6), interleukin 8 (IL8/CXCL8), interleukin 10 (IL10), tumour necrosis Factor—alpha (TNFα), interferon gamma (IFNγ), chemokine (C-C motif) ligand 2 (CCL2/MCP1), chemokine ligand 5 (CCL5/RANTES), chemokine (C-X-C motif) ligand 9 (CXCL9/MIG), and chemokine ligand 10 (CXCL10/IP10) measured as described below. Secondary outcome measures were PaO_2_/FiO_2_ ratios at 6, 12 and 24 hours post-transplant.

### Statistics

Statistical analysis was performed in SPSS Statistics 22 (IBM, Chicago, USA) and graphed in GraphPad Prism 6.01 (GraphPad, California, USA). Analyses conducted were linear regressions, mixed model linear repeated measures (RIC or sham treatment, diagnosis) with an unstructured repeated covariance design, and one-way ANOVAs (diagnosis). Bonferroni post-hoc tests were used as appropriate. To correct for multiple comparisons, Bonferonni’s correction was applied. Proportions in Table 1 (patient characteristics) were compared using a Chi square test, with exact statistics calculated. Other comparisons in Table 1 used a Student’s t-test. Bootstrapping with 10,000 samples was used to generate 95% confidence intervals. Data presented as mean ± SEM. P<0.05 is regarded as statistically significant.

**Table 1:** Summary of patient and donor characteristics stratified by ischemic conditioning or sham treatment.

	RIC (n = 24)	Sham Treatment (n = 22)	p-value (RIC vs Sham)	Patient Numbers (n = 46)
Sex	Female: 13/Male: 11	Female: 10/Male: 12	0.768	Female: 23/Male: 23
Diagnostic Group	Obst. CF: 8	Obst. CF: 4	0.497	Obst. CF: 12
	Obst. Non-CF: 12	Obst. Non-CF: 13		Obst. Non-CF: 25
	Restrictive: 4	Restrictive: 5		Restrictive: 9
Age (Years)	49.54±2.54 (95%CI: 44.64–54.66)	51.11±2.85 (95%CI: 45.44–56.62)	0.689	50.29±1.93 (95%CI: 46.27–53.95)
ICU Length of Stay (Days)	8.46±2.06 (95%CI: 5.19–13.26)	7.77±1.87 (95%CI: 4.68–11.86)	0.811	8.13±1.39 (95%CI: 5.81–11.02)
Total Length of Stay (Days)	21.54±2.20 (95%CI: 17.96–26.54)	25.41±3.42 (95%CI: 19.63–33.36)	0.347	23.39±2.06 (95%CI: 19.91–27.74)
Ischemic Time for 1st Lung (Mins)	241.25±15.86 (95%CI: 211.00–274.90)	242.05±21.45 (95%CI: 202.68–286.68)	0.977	241.63±13.30 (95%CI: 218.33–269.50)
Ischemic Time for 2nd Lung (Mins)	352.75±19.24 (95%CI: 314.62–391.64)	336.41±21.91 (95%CI: 295.19–382.23)	0.577	344.93±14.87 (95%CI: 316.86–372.67)
Patient Death by end of follow-up	Alive: 17/Dead: 7	Alive: 19/Dead: 3	0.289	Alive: 36/Dead: 10
Donor Status	After cardiac death: 7	After cardiac death: 9	0.538	After cardiac death: 16
	After brain death: 17	After brain death: 13		After brain death: 30
Donor pO2 (mmHg)	431.92±21.97 (95%CI: 384.60–471.50)	462.77±13.62 (95%CI: 436.35–491.41)	0.259	446.67±12.70 (95%CI: 420.96–472.85)

### Data transformation and missing values analysis

Some results >6 SD from the mean (6 data points: 1 IL6, 2 CCL2, and 3 CXCL8) were set to missing. Data was otherwise not transformed or modified prior to, during or after analysis. Missing data was not replaced and was randomly distributed across time; however, certain assays had high rates of missing values due to the multiplex assay kit (TNFα, IFNλ, IL2 and IL4). Missing partial pressure of arterial oxygen/fraction of inspired oxygen ratios (PF) ratios result from patients being extubated after which arterial catherization was no longer available. N values should be noted when interpreting results. Missing data analysis demonstrated that multiple imputation, linear interpolation and mean of nearby points would not reliably estimate missing data, given the relatively few patients in this study. Therefore, we used a mixed model linear repeated measures design with an unstructured repeated covariance design, as noted above, enabling use of all available data in the model.

The data set needed for replication have been uploaded to Figshare and are available through the following link: http://figshare.com/article/Substantial increases occur in serum Activins and Follistatin during lung transplantation /1547913

## Results

### Patient and Donor Characteristics

There were no statistically significant differences between groups who received RIC or the sham treatment (Table 1). RIC did not change the proportion of patients alive at the end of follow-up, the length of time patients spent in intensive care, or total hospital stay length. Details of the diagnostic groups comprising the patient cohort are provided in Table 1.

### Effect of remote ischemic conditioning on PiO2/FiO2 ratios measured at 6, 12 and 24 hours

As published previously, RIC did not affect PiO2/FiO2 ratios [24].

### Serum Activin A concentrations compared to reference values

**Fig 1:**
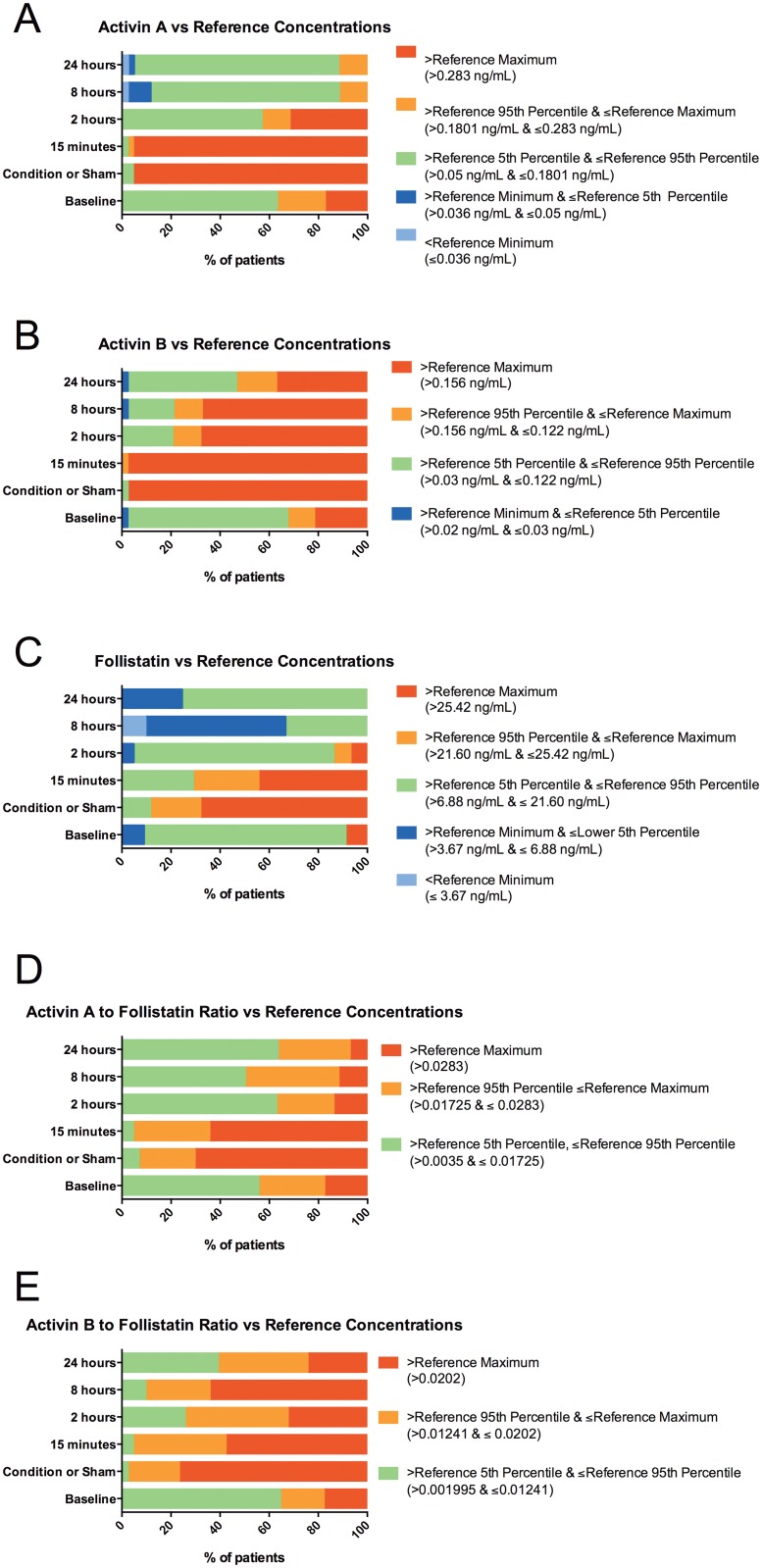
**(A)** Serum activin A levels for the patients are illustrated and compared to the upper and lower reference thresholds for normal healthy volunteers (25). **(B)** Serum activin B levels for the patients are illustrated and compared to the upper and lower reference thresholds for normal healthy volunteers (25). **(C)** Serum follistatin levels for the patients are illustrated and compared to the upper and lower reference thresholds for normal healthy volunteers (25). **(D)** Serum Activin A to follistatin ratios for the patients are compared with the upper and lower reference ratio thresholds for normal healthy volunteers (25). **(E)** Serum Activin B to follistatin ratios for the patients are compared with the upper and lower reference ratios for normal healthy volunteers (25).

Baseline serum activin A concentrations were above the reference maximum (RM) (>0.283ng/mL) in 17.4% of patients (Fig 1A). The levels rose significantly, peaking in the RIC/Sham time point sample, which immediately preceded reperfusion of the first lung. Activin A levels at RIC/Sham time-point were above the RM in 95.6% of patients. This increase was sustained at 15 minutes post-transplant reperfusion, with 95.7% of patients having activin A concentrations above the RM declining further with only 31.8% of patients remaining above the RM at 2 hours post-transplant and by 8 hours activin A levels were normal in all patients.

### Serum activin B concentrations compared to reference values

Baseline serum activin B was above the RM concentration (>0.156ng/mL) in 21.7% of patients (Fig 1B) The levels increased at the RIC/sham treatment and 15 minutes post-transplant time-points; only one patient remaining below the RM. The serum activin B levels remained elevated above the RM in 68.2% of patients at 2 hours, in 67.4% at 8 hours and in 37.2% of patients at 24 hours.

### Serum follistatin concentrations compared to reference values

Baseline serum FS levels were above the RM (>25.42ng/mL) in 8.7% of patients (Fig 1C). The FS levels increased in the sample taken after the RIC/sham treatment, with the levels in 68.9% of patients being above the RM. At 15 minutes post-transplant reperfusion, 43.5% of patients were above the RM and decreased further with only 3 patients having levels above the RM at 2 hours post-transplant. FS levels were within the normal range in all patients subsequently.

### Ratio of activin A to follistatin concentrations, a marker of activin A bioactivity, compared to reference values

The ratio of activin A to FS, an established surrogate measure of activin bioactivity, was greater than the RM ratio (>0.0283) in 17.4% of patients at baseline (Fig 1D). After the RIC/sham treatment, the levels in 71.1% of patients were above normal and remained elevated at 15 minutes post-transplant in 63% of patients. The levels decreased subsequently and at 2 hours post-transplant only 13.6% of patients had elevated levels, decreasing to 11.6% at 8 hours and to 7.1% at 24 hours post-transplant. No patient remained above normal for the whole study.

### Ratio of activin B to follistatin concentrations, a marker of activin B bioactivity, compared to reference values

Activin B to FS ratios were greater than normal in 17.4% of patients at baseline (Fig 1E) and increased after RIC/sham treatment, with 77% of patients above normal. This proportion decreased to 58.7% above normal at 15 minutes post-transplant, to 34.1% at 2 hours post-transplant, increasing at 8 hours post-transplant to 66.7% but by 24 hours post-transplant had decreased to 26.2% of patients above RM. Three patients remained above normal throughout the study period.

### Correlations between peak values

The peak activin A level and peak activin B level from each patient were not correlated (p = 0.810). Peak activin A and peak FS levels from each patient were correlated (r^2^ = 0.228, p = 0.0005) as were peak activin B and peak FS levels (p<0.0001).

There were no significant correlations between the lowest PiO2/FiO2 ratio from each patient (6, 12 and 24 hour time-points) and any of the following measurements, peak serum activin A concentration (p = 0.749), peak serum activin B concentration (p = 0.104), peak serum FS concentration (p = 0.208), peak activin A/FS ratio (p = 0.315), and peak activin B/FS ratio (p = 0.594).

### Correlations between cytokines

It is not possible to report all cytokine correlations, so we present correlations that were consistent across multiple time-points and therefore are likely to be biologically relevant in Tables 2 and 3. Activin A and B levels were mostly correlated across time, apart from after the RIC/sham treatment and 15 minutes post-transplant. Activin A, activin B and FS were consistently correlated across all time-points, except for activin B and FS at 8 hours post-transplant. Details concerning all the correlations between activins A, activin B and follistatin and all the other cytokines measured are given in S1 Table.

**Table 2:** Correlations between activins and follistatin.

Factor	Divisor	Time-point	n	Adj R^2^	p-value	Factor	Divisor	Time-point	n	Adj R^2^	p-value
Activin A	Activin B	Baseline	46	0.796	<0.0001	Activin A	CXCL10	Baseline	46	0.032	0.121
		Condit./Sham	44	-0.017	0.597			Condit./Sham	45	0.057	0.063
		15 minutes	46	0.012	0.299			15 minutes	46	0.114	0.013
		2 hours	44	0.346	<0.0001			2 hours	43	0.069	0.049
		8 hours	43	0.344	<0.0001			8 hours	43	0.157	0.005
		24 hours	42	0.323	<0.0001			24 hours	42	-0.022	0.746
Activin A	FS	Baseline	46	0.685	<0.0001	Activin B	CXCL8	Baseline	46	0.005	0.272
		Condit./Sham	45	0.318	<0.0001			Condit./Sham	44	-0.023	0.901
		15 minutes	46	0.389	<0.0001			15 minutes	46	0.011	0.230
		2 hours	44	0.444	<0.0001			2 hours	43	-0.019	0.635
		8 hours	43	0.013	0.222			8 hours	43	0.296	<0.0001
		24 hours	42	0.267	0.00027			24 hours	42	-0.021	0.688
Activin B	FS	Baseline	46	0.788	<0.0001	Activin B	CXCL9	Baseline	46	0.103	0.017
		Condit./Sham	44	0.300	<0.0001			Condit./Sham	44	0.079	0.036
		15 minutes	46	0.360	<0.0001			15 minutes	46	0.229	0.0004
		2 hours	44	0.850	<0.0001			2 hours	43	0.046	0.091
		8 hours	43	-0.024	0.896			8 hours	43	-0.024	0.992
		24 hours	42	0.210	0.0014			24 hours	42	-0.025	0.931
Activin A	CXCL8	Baseline	46	0.076	0.036	Activin B	CXCL10	Baseline	46	-0.007	0.407
		Condit./Sham	45	0.044	0.089			Condit./Sham	44	-0.023	0.850
		15 minutes	46	-0.021	0.764			15 minutes	46	-0.018	0.660
		2 hours	43	-0.019	0.655			2 hours	43	0.075	0.042
		8 hours	43	0.252	0.0004			8 hours	43	-0.014	0.525
		24 hours	42	0.107	0.020			24 hours	42	-0.021	0.687
Activin A	CXCL9	Baseline	46	0.252	0.00023						
		Condit./Sham	45	0.101	0.019						
		15 minutes	46	0.162	0.003						
		2 hours	43	0.096	0.024						
		8 hours	43	0.200	0.002						
		24 hours	42	0.014	0.213						

**Table 3:** Correlations between activin to follistatin ratios, and other cytokines, and between other cytokines.

Factor	Divisor	Time-point	n	Adj R^2^	p-value	Factor	Divisor	Time-point	n	Adj R^2^	p-value
Act.A to FS Ratio	CXCL8	Baseline	46	0.151	0.004	Act.B to FS Ratio	CXCL10	Baseline	46	-0.022	0.824
		Condit./Sham	45	0.023	0.160			Condit./Sham	44	0.031	0.132
		15 minutes	46	-0.021	0.799			15 minutes	46	0.028	0.137
		2 hours	43	-0.007	0.404			2 hours	43	0.056	0.068
		8 hours	43	-0.005	0.385			8 hours	43	-0.013	0.500
		24 hours	42	-0.018	0.598			24 hours	42	0.005	0.280
Act.A to FS Ratio	CXCL9	Baseline	46	0.177	0.002	CXCL8	CXCL10	Baseline	46	-0.013	0.511
		Condit./Sham	45	0.043	0.093			Condit./Sham	45	0.157	0.004
		15 minutes	46	0.027	0.140			15 minutes	46	0.037	0.107
		2 hours	43	0.111	0.016			2 hours	43	-0.007	0.400
		8 hours	43	0.049	0.083			8 hours	43	0.231	0.001
		24 hours	42	-0.025	0.920			24 hours	42	0.034	0.125
Act.A to FS Ratio	CXCL10	Baseline	46	0.034	0.114	CXCL8	CXCL9	Baseline	46	-0.020	0.737
		Condit./Sham	45	0.000	0.319			Condit./Sham	45	-0.023	0.896
		15 minutes	46	0.011	0.227			15 minutes	46	-0.016	0.582
		2 hours	43	0.132	0.010			2 hours	43	-0.023	0.841
		8 hours	43	0.000	0.318			8 hours	43	0.032	0.130
		24 hours	42	0.005	0.280			24 hours	42	0.038	0.113
Act.B to FS Ratio	CXCL8	Baseline	46	0.058	0.058	CXCL9	CXCL10	Baseline	46	0.411	<0.0001
		Condit./Sham	44	0.006	0.271			Condit./Sham	45	0.278	0.00012
		15 minutes	46	0.121	0.010			15 minutes	46	0.226	0.0005
		2 hours	43	0.086	0.031			2 hours	43	0.457	<0.0001
		8 hours	43	-0.014	0.522			8 hours	43	0.499	<0.0001
		24 hours	42	-0.018	0.598			24 hours	42	0.449	<0.0001
Act.B to FS Ratio	CXCL9	Baseline	46	0.064	0.049						
		Condit./Sham	44	0.001	0.309						
		15 minutes	46	0.037	0.106						
		2 hours	43	0.029	0.139						
		8 hours	43	-0.022	0.756						
		24 hours	42	-0.025	0.920						

### Effect of diagnostic grouping on serum activin A and B, follistatin and other cytokines

**Fig 2:**
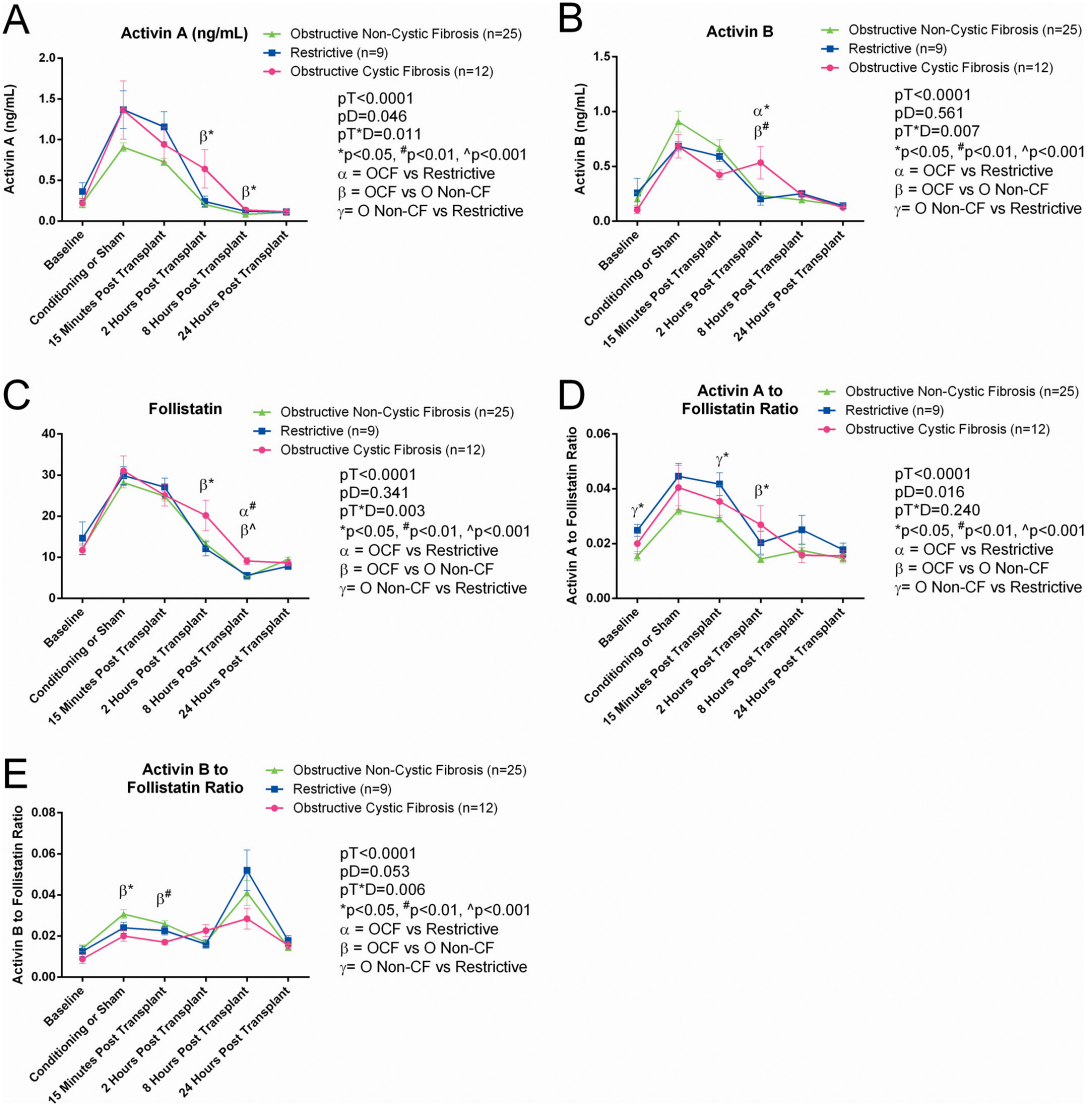
The serum levels of the activin A. (A), activin B (B), follistatin (C), activin A to follistatin ratio (D), and activin B to follistatin ratio (E) measured over the 24 hour time course of this study are illustrated with the patients characterized according to a pathological classification based on the primary cause of their lung disorder.

Serum activin A concentrations were significantly affected by diagnostic grouping and by diagnostic group across time (Fig 2A). Post-hoc analysis revealed that serum activin A levels at both 2 hours and 8 hours post-transplant were significantly greater in patients with obstructive CF than in obstructive non-CF patients compared to obstructive-non-CF (p = 0.017).

Serum activin B concentrations differed across time with diagnostic group as a factor (Fig 2B). At 2 hours post-transplant, patients with obstructive CF had significantly higher concentrations than those with obstructive non-CF (p = 0.017) vs obstructive CF) and patients with restrictive disease (p = 0.049).

Follistatin concentrations were affected by diagnostic group over time (Fig 2C). At 2 hours post-transplant FS levels were elevated in patients with obstructive CF compared to those with obstructive non-CF (p = 0.05). At 8 hours post-transplant, FS was still elevated in the obstructive CF group, compared with both obstructive non-CF (p<0.0001) and restrictive pulmonary disease (p = 0.001).

Across the 24 hrs, activin A to FS ratios were significantly different between diagnostic groups, patients with restrictive pulmonary disease had significantly higher ratios compared with obstructive non-CF (p = 0.021) (Fig 2D). Post-hoc analysis demonstrated that basal ratios were elevated significantly (p = 0.04) in patients with restrictive disease compared to those with obstructive non-CF. These remained significantly elevated at 15 minutes post-transplant (p = 0.036) and at 2 hours post-transplant (p = 0.042).

The activin B to FS ratio was not quite significantly (p = 0.053) different between diagnostic groups (Fig 2E). At the conditioning/sham time-point, ratios in patients with obstructive non-CF were elevated compared with obstructive CF (p = 0.015). This continued at 15 minutes post-transplant (p = 0.002).

The diagnostic grouping of patients identified significant correlations with some of the other cytokines (S1 Fig). There was no effect of diagnostic group on serum concentrations of IL2, IL4, IL6, TNFα, INFγ, CXCL9/MIG, CCL2/MCP2 or CXCL10/IP10.

IL10 (S1D Fig) was elevated significantly (p = 0.045) at the conditioning/sham time-point in patients with obstructive CF compared to those with obstructive non-cystic fibrosis and restrictive disease (p = 0.045) (S1 Fig). CXCL8/IL8 was elevated at the conditioning/sham time-point only in patients with obstructive CF compared with patients with obstructive non-CF (p = 0.005).

The CCL5/RANTES (S2B Fig) levels on average across the 24 hours, were significantly elevated in patients with obstructive CF compared with obstructive non-CF (S1 and S2 Figs) (obstructive CF: 9525.06 ± 1023.33 n = 12; obstructive non-CF: 5559.81 ± 708.81 n = 25; p = 0.008) (S2 Fig). This continued with post-hoc analysis at baseline (p = 0.020), 2 hours post-transplant (p = 0.001) and 8 hours post-transplant (p = 0.047).

The serum levels of CXCL8-IL8 (A), CCL5 RANTES (B), CXCL9 MIG (C), CCL2 MCP1 (D), and TNF (E), measured over the 24 hour time course of this study, showed a significantly decreased response to the transplantation surgery in the CXCL 9 and CXCL10 response in patients undergoing RIC but no other significant changes (S1 and S2 Figs)

### Effect of RIC on serum concentrations of activins, follistatin and other cytokines

**Fig 3:**
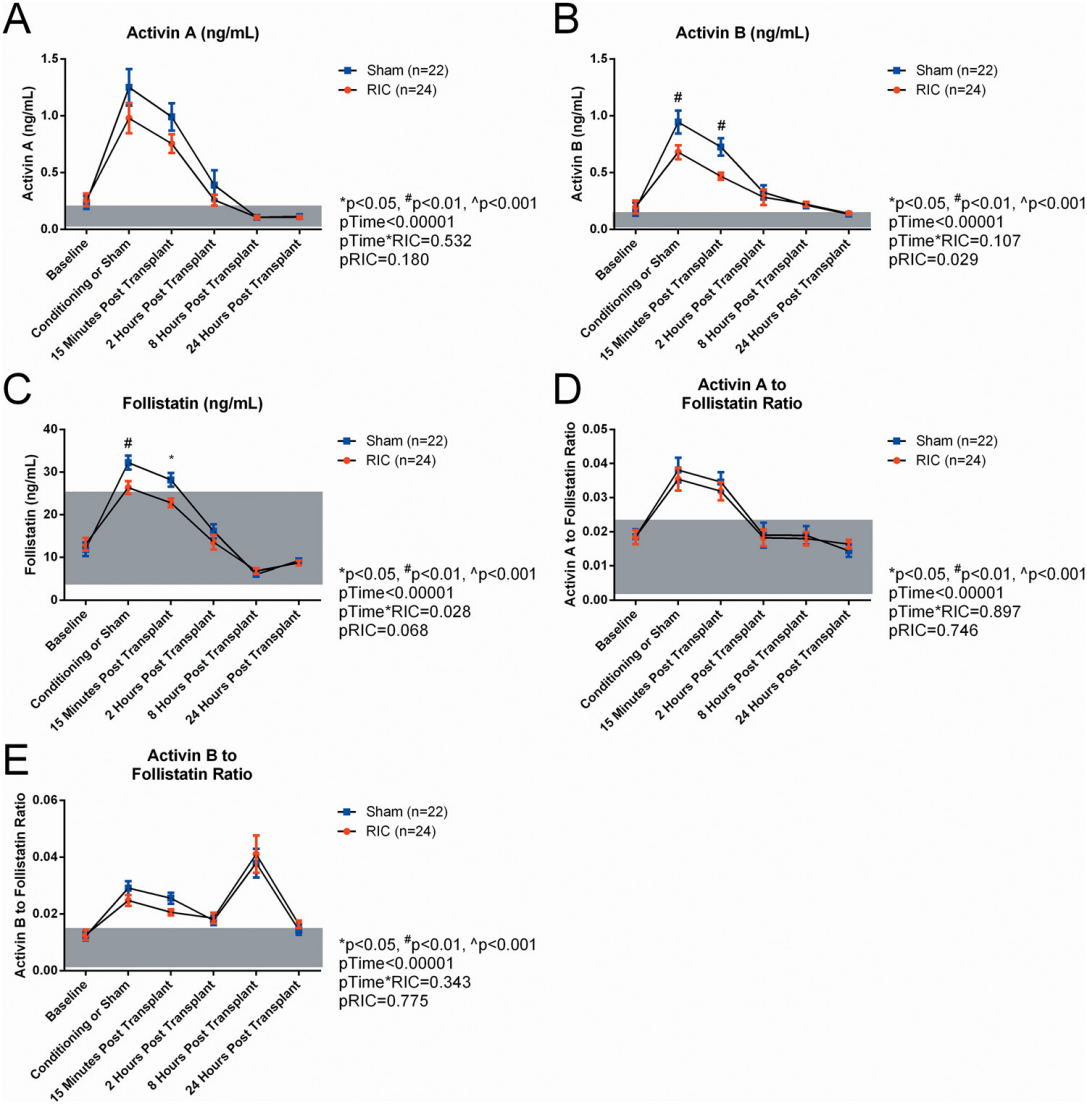
Serum concentrations of activin A. (A), activin B (B), follistatin (C), activin A/follistatin ratio (D) and activin B/follistatin ratio (E), stratified by whether the patient received RIC or were in the sham treatment group. Grey boxes are the minimum to maximum reference range for each analyte and ratio.

RIC did not change serum activin A across time or at any individual time-point (Fig 3A) but reduced the average serum activin B levels over 24 hours (RIC: 0.33 ± 0.03, n = 24, Sham: 0.42 ± 0.030 n = 22p = 0.029; Fig 3B) and post-hoc analysis showed this was primarily due to decreased serum levels in the RIC exposed patients at the condition/sham time-point (p = 0.033) and the 15 minute time-point (p = 0.003) (Fig 3B). There was also a significant decrease in the response of CXCL8 and CXCL 10 in the RIC treated patients (S4 Fig).

The 24 hour average serum FS level was also decreased by 15% in RIC treated compared with sham treated patients (p = 0.047) (Fig 3C). Comparing RIC treated with sham patients, there was an 18% reduction at the conditioning/sham time-point in RIC treated patients compared with sham treated patients (p = 0.010), and a 20.1% reduction at 15 minutes post-transplant (p = 0.003).

The ratio of activin A to FS and activin B to FS were mostly unaffected by RIC treatment or sham treatment (Fig 3D and 3E). RIC significantly lowered the activin B to FS ratio by 19% compared with sham treated patients only at the 15-minute post-transplant time point (p = 0.037) (Fig 3E).

RIC had no significant effect on the following cytokines: IL2, IL4, IL6, IL10, TNFα, INFγ, CXCL8/IL8, CCL5 RANTES, CCL2/MCP1 and CXCL10/IP10 (S3 and S4 Figs). CXCL9/MIG was 46.2% lower in RIC compared to sham treated patients at the conditioning/sham time-point (p = 0.016) and 48% lower in RIC patients at 15 minutes post-transplant (p = 0.019) (S4 Fig).

### Predictors of PiO2/FiO2 ratios measured at 6, 12 and 24 hours

In this analysis data included for each time point only includes data collected prior to that time point. Factors correlated with PiO2/FiO2 at 6 hours were ischemic time for the 1^st^ lung (p = 0.006), ischemic time for the 2^nd^ lung (p = 0.011), serum activin B measured at conditioning/sham (p = 0.011), and the activin B to FS ratio measured at the conditioning/sham time-point (p = 0.006).

At 12 hours the factors were exact recipient age (p = 0.034), activin B level at the conditioning/sham time-point (p = 0.018), activin B to FS ratio measured at the conditioning/sham time-point (p = 0.028), IL4 measured at the conditioning/sham time-point (p = 0.018), IL6 measured at the 8 hours post-transplant time-point (p = 0.020), and CCL5/RANTES measured at the 8 hours post-transplant time-point (p = 0.032). At 24 hours post-transplant the only factor that correlated with PiO2/FiO2 was the IL4 level at 15 minutes post-transplant (p = 0.039).

Given the low number of cases and sparse direct associations across time, further modelling was not attempted as results would almost certainly not be generalizable to other patient populations.

## Discussion

This study establishes that substantial increases in serum concentrations of activin A, B and FS occur during lung transplantation surgery. Since activin A is a key cytokine regulating pro-inflammatory and pro-fibrotic processes [27, 28], this study raises the possibility that the substantial increases in activin A and B could compromise the function of transplanted lungs. Only further long term functional studies will answer this concern and determine if there is a need to change procedures, medications or other interventions that could decrease the magnitude of these increases in activins A and B.

The increases in basal activin A and B levels in these patients likely arise from the pathophysiological processes causing the need for lung transplantation. Both clinical and animal studies demonstrate that high activin A levels cause inflammation and the resultant fibrosis compromises lung function [27, 29]. Elevated serum activin A levels occur in patients with a variety of inflammation-based lung disorders such as CF, bleomycin-induced fibrosis, allergy-based inflammatory models and idiopathic pulmonary fibrosis [27–30] and are consistent with the capacity of elevated activin A levels to cause hepatic and renal fibrosis [16, 31].

The rapid increase in serum activin A concentrations associated with lung transplantation partly reflects the increase triggered by the acute phase response to surgery [18, 32, 33]. The same mechanisms are likely to be the cause of the novel finding of elevated serum activin B levels. Given that the magnitude of the increases of activin A and B surpass those associated with the expected acute phase response, we suspected that other factors were involved.

The most likely cause of the magnitude of the rapid increase in activin A and B is the use of heparin anti-coagulation. 5,000–10,000 units of heparin are administered to the transplant recipient shortly after anaesthesia induction. The transplant donor also receives a substantial dose of heparin during transplant donation (up to 25,000IU given to brain dead donors), and additional heparin is administered to the transplanted organ (up to 25,000IU).

In sheep, we demonstrated that heparin injections led to a rapid dose-related increase in serum activin A and FS [20] due to the negatively charged heparin releasing FS and activin A bound to cell surface proteoglycans via a series of positively charged amino acids (arginine) in the FS sequence [19]. This binding site is also the mechanism by which FS-bound activin A is targeted to lysosomal degradation [15, 34]. The capacity of heparin to release FS and activin A was also shown in patients undergoing cardiac procedures using heparin [21, 22]. Using a sheep model of cardiopulmonary bypass, Chen et al [21] showed that increases in serum activin A and FS did not occur when non-heparin anticoagulants were used. It is possible that the magnitude of the increase in activin A is in fact greater since all patients received significant doses of methyl prednisolone as a prelude to transplantation. Previous studies have shown that glucocorticoids will decrease activin A levels [11].

We propose that heparin use and the acute phase response are the likely cause of the novel finding that serum activin B levels also increase at this time point. Further studies are necessary to determine the pathophysiological actions of the elevated activin B concentrations. While the early functional capacity of the transplanted lung did not show any correlation with the magnitude of the activin A and B released during transplant surgery, further long term studies are needed to clarify this issue.

RIC significantly decreased the 24 hour average levels of activin B and FS, and seemed to blunt the maximal rise of activin B and FS. Further, the area under the curve in Fig 3, representing the 24 hour activin A levels are lower but not significantly and when combined with the decrease in activin B post-RIC, may explain the lower levels of FS during the same period, as both activins are determinants of FS production [11,18]. However, these changes did not affect the lowest P/F ratio or the average P/F ratio across the 24 hours. These findings warrant a larger study of RIC will be required to confirm its effects on the release profile of activin A, B and FS. Apart for activins A and B, the only other cytokine to be affected by RIC was CXCL9 that shows a marked decrease in response to RIC.

We note that of all the cytokines measured, the activins A and B are consistently the two cytokines which increase very early in lung transplantation surgery, a pattern similar to the activin A response to LPS [13]. These observation support the view that activin A rapidly responds to the IRI during the initial reperfusion of the transplanted lung and continues in those patients randomized to receive RIC and is the likely initiator of the inflammatory response [13]. As discussed earlier, the magnitude of this response is augmented by the use of heparin. Further studies are required to determine if activin B also has the capability to initiate inflammatory responses [13]. Since 1μg of FS, given 1 hour prior to an LPS challenge, by binding and neutralizing the actions of activin A, markedly changed the cytokine response to LPS such as halving the release of TNFα, emphasizes the capacity of activin A to act as a master controller of the cytokine response. The rapid response of both activin A and B to IRI also supports the concept that they are the “initiators” of this response. As such, changing the type of anti-coagulant used could substantially reduce the release of the activins. Alternatively, administering FS just prior and during the reperfusion of a transplant could minimize any resultant functional impairment of the transplant.

The peak activin A levels released during lung transplantation were of greater magnitude than those associated with death in patients with septicaemia or with higher mortality in patients in intensive care units with acute respiratory failure [25, 35]. It is possible that the concomitant release of FS may protect the transplant and other tissues from the damaging actions of activin A that include apoptosis of hepatocytes and B lymphocytes [36, 37]. The latter may affect the morbidity associated with transplantation surgery, outcomes that could be clarified by comparing the use of heparin and non-heparin based anti-coagulants.

As heparin is administered to most donors prior to organ collection for transplantation, the use of non-heparin anticoagulants offers a simple way of resolving this issue. Alternatively, administration of FS to the donor at lung collection or adding it to preservation solutions may decrease organ damage. Further, FS use during perfusion of lungs on a rig prior to transplantation may also be worthy of assessment to preserve lung function [6].

The large increases in activin A and B during transplant surgery are clearly influenced by many factors including the cause of the patient’s lung injury, the acute phase response and the dose of heparin administered during the entire transplant surgery. Further studies to assess the effects of heparin versus non-heparin anti-coagulants during lung transplantation will clarify these issues and determine if a change in anti-coagulant practice can improve patient outcomes.

## Supporting Information

S1 Fig Changes in the serum levels of IL2, IL4, IL6, IL10, TNFα and INFλ during lung transplantation surgery in patients with obstructive non-cystic fibrosis lung disease, obstructive cystic fibrosis lung disease and restrictive lung disease are illustrated. (TIF)

S2 Fig Changes in the serum levels of CXCL8, CCL5, CXCL9, CCL2 and CXCL10 during lung transplantation surgery in patients with obstructive non-cystic fibrosis lung disease, obstructive cystic fibrosis lung disease and restrictive lung disease are illustrated. (TIF)

S3 Fig The concentrations of serum IL2 (A), IL4 (B), IL6 (C), IL10 (D), TNFα (E) and Interferon Gamma (F) measured are illustrated by grouping according to whether the patient received RIC or were in the sham treatment group. (TIF)

S4 Fig The concentrations of the serum CXCL8-IL8 (A), CCL5 RANTES (B), CXCL9 MIG (C), CCL2 MCP1 (D), CXCL10 IP10 (E) measured are illustrated by grouping according to whether the patient received RIC or were in the sham treatment group. Note a significantly lower response following RIC in the serum levels of CXCL9(MIG) and CXCL10(IP10).
(TIF)

S1 Table The correlations between the activin A, activin B and follistatin levels, their ratios and all the other cytokines measured are detailed in S1 Table. (DOCX)
