# Supplementary material for: Substantial Increases Occur in Serum Activins and Follistatin during Lung Transplantation
Source: PLoS One. 2016 Jan 28;11(1):e0140948. doi: 10.1371/journal.pone.0140948 (PMC4731072; doi:10.1371/journal.pone.0140948)
Supplement: S1 Table — (DOCX) [file pone.0140948.s005.docx]

**S1 Table**: Correlations between activin to follistatin ratios, and other cytokines, and between other cytokines

| Factor | Divisor | Time-point | n | Adj R^2^ | p-value |  | Factor | Divisor | Time-point | n | Adj R^2^ | p-value |
| --- | --- | --- | --- | --- | --- | --- | --- | --- | --- | --- | --- | --- |
| Act.A to FS Ratio | CXCL8 | Baseline | 46 | 0.151 | 0.004 |  | Activin A | CXCL10 | Baseline | 46 | 0.032 | 0.121 |
|  |  | Condit./Sham | 45 | 0.023 | 0.160 |  |  |  | Condit./Sham | 45 | 0.057 | 0.063 |
|  |  | 15 minutes | 46 | -0.021 | 0.799 |  |  |  | 15 minutes | 46 | 0.114 | 0.013 |
|  |  | 2 hours | 43 | -0.007 | 0.404 |  |  |  | 2 hours | 43 | 0.069 | 0.049 |
|  |  | 8 hours | 43 | -0.005 | 0.385 |  |  |  | 8 hours | 43 | 0.157 | 0.005 |
|  |  | 24 hours | 42 | -0.018 | 0.598 |  |  |  | 24 hours | 42 | -0.022 | 0.746 |
| Act.A to FS Ratio | CXCL9 | Baseline | 46 | 0.177 | 0.002 |  | Activin B | CXCL8 | Baseline | 46 | 0.005 | 0.272 |
|  |  | Condit./Sham | 45 | 0.043 | 0.093 |  |  |  | Condit./Sham | 44 | -0.023 | 0.901 |
|  |  | 15 minutes | 46 | 0.027 | 0.140 |  |  |  | 15 minutes | 46 | 0.011 | 0.230 |
|  |  | 2 hours | 43 | 0.111 | 0.016 |  |  |  | 2 hours | 43 | -0.019 | 0.635 |
|  |  | 8 hours | 43 | 0.049 | 0.083 |  |  |  | 8 hours | 43 | 0.296 | <0.0001 |
|  |  | 24 hours | 42 | -0.025 | 0.920 |  |  |  | 24 hours | 42 | -0.021 | 0.688 |
| Act.A to FS Ratio | CXCL10 | Baseline | 46 | 0.034 | 0.114 |  | Activin B | CXCL9 | Baseline | 46 | 0.103 | 0.017 |
|  |  | Condit./Sham | 45 | 0.000 | 0.319 |  |  |  | Condit./Sham | 44 | 0.079 | 0.036 |
|  |  | 15 minutes | 46 | 0.011 | 0.227 |  |  |  | 15 minutes | 46 | 0.229 | 0.0004 |
|  |  | 2 hours | 43 | 0.132 | 0.010 |  |  |  | 2 hours | 43 | 0.046 | 0.091 |
|  |  | 8 hours | 43 | 0.000 | 0.318 |  |  |  | 8 hours | 43 | -0.024 | 0.992 |
|  |  | 24 hours | 42 | 0.005 | 0.280 |  |  |  | 24 hours | 42 | -0.025 | 0.931 |
| Act.B to FS Ratio | CXCL8 | Baseline | 46 | 0.058 | 0.058 |  | Activin B | CXCL10 | Baseline | 46 | -0.007 | 0.407 |
|  |  | Condit./Sham | 44 | 0.006 | 0.271 |  |  |  | Condit./Sham | 44 | -0.023 | 0.850 |
|  |  | 15 minutes | 46 | 0.121 | 0.010 |  |  |  | 15 minutes | 46 | -0.018 | 0.660 |
|  |  | 2 hours | 43 | 0.086 | 0.031 |  |  |  | 2 hours | 43 | 0.075 | 0.042 |
|  |  | 8 hours | 43 | -0.014 | 0.522 |  |  |  | 8 hours | 43 | -0.014 | 0.525 |
|  |  | 24 hours | 42 | -0.018 | 0.598 |  |  |  | 24 hours | 42 | -0.021 | 0.687 |
| Act.B to FS Ratio | CXCL9 | Baseline | 46 | 0.064 | 0.049 |  | CXCL8 | CXCL10 | Baseline | 46 | -0.013 | 0.511 |
|  |  | Condit./Sham | 44 | 0.001 | 0.309 |  |  |  | Condit./Sham | 45 | 0.157 | 0.004 |
|  |  | 15 minutes | 46 | 0.037 | 0.106 |  |  |  | 15 minutes | 46 | 0.037 | 0.107 |
|  |  | 2 hours | 43 | 0.029 | 0.139 |  |  |  | 2 hours | 43 | -0.007 | 0.400 |
|  |  | 8 hours | 43 | -0.022 | 0.756 |  |  |  | 8 hours | 43 | 0.231 | 0.001 |
|  |  | 24 hours | 42 | -0.025 | 0.920 |  |  |  | 24 hours | 42 | 0.034 | 0.125 |
| Act.B to FS Ratio | CXCL10 | Baseline | 46 | -0.022 | 0.824 |  | CXCL8 | CXCL9 | Baseline | 46 | -0.020 | 0.737 |
|  |  | Condit./Sham | 44 | 0.031 | 0.132 |  |  |  | Condit./Sham | 45 | -0.023 | 0.896 |
|  |  | 15 minutes | 46 | 0.028 | 0.137 |  |  |  | 15 minutes | 46 | -0.016 | 0.582 |
|  |  | 2 hours | 43 | 0.056 | 0.068 |  |  |  | 2 hours | 43 | -0.023 | 0.841 |
|  |  | 8 hours | 43 | -0.013 | 0.500 |  |  |  | 8 hours | 43 | 0.032 | 0.130 |
|  |  | 24 hours | 42 | 0.005 | 0.280 |  |  |  | 24 hours | 42 | 0.038 | 0.113 |
| Activin A | CXCL8 | Baseline | 46 | 0.076 | 0.036 |  | CXCL9 | CXCL10 | Baseline | 46 | 0.411 | <0.0001 |
|  |  | Condit./Sham | 45 | 0.044 | 0.089 |  |  |  | Condit./Sham | 45 | 0.278 | 0.00012 |
|  |  | 15 minutes | 46 | -0.021 | 0.764 |  |  |  | 15 minutes | 46 | 0.226 | 0.0005 |
|  |  | 2 hours | 43 | -0.019 | 0.655 |  |  |  | 2 hours | 43 | 0.457 | <0.0001 |
|  |  | 8 hours | 43 | 0.252 | 0.0004 |  |  |  | 8 hours | 43 | 0.499 | <0.0001 |
|  |  | 24 hours | 42 | 0.107 | 0.020 |  |  |  | 24 hours | 42 | 0.449 | <0.0001 |
| Activin A | CXCL9 | Baseline | 46 | 0.252 | 0.00023 |  |  |  |  |  |  |  |
|  |  | Condit./Sham | 45 | 0.101 | 0.019 |  |  |  |  |  |  |  |
|  |  | 15 minutes | 46 | 0.162 | 0.003 |  |  |  |  |  |  |  |
|  |  | 2 hours | 43 | 0.096 | 0.024 |  |  |  |  |  |  |  |
|  |  | 8 hours | 43 | 0.200 | 0.002 |  |  |  |  |  |  |  |
|  |  | 24 hours | 42 | 0.014 | 0.213 |  |  |  |  |  |  |  |
